# Quality of life and ability to work of patients with Post-COVID syndrome in relation to the number of existing symptoms and the duration since infection up to 12 months: a cross-sectional study

**DOI:** 10.1007/s11136-023-03369-2

**Published:** 2023-03-03

**Authors:** Christina Lemhöfer, Christian Sturm, Dana Loudovici-Krug, Christoph Guntenbrunner, Marcus Bülow, Philipp Reuken, Stefanie Quickert, Norman Best

**Affiliations:** 1 Institute of Physical and Rehabilitation Medicine, University Hospital Jena, Am Klinikum 1, 07743 Jena, Germany; 2 Hannover Medical School, Clinic for Rehabilitation Medicine, Hannover, Germany; 3 Clinic for Internal Medicine IV (Gastroenterology, Hepatology, Infectiology), University Hospital Jena, Jena, Germany

**Keywords:** Health-related quality of Life, Long-term complication, Work ability, COVID-19

## Abstract

**Purpose:**

Following SARS-CoV-2 virus infection, patients may suffer from long-lasting symptoms regardless of disease severity. Preliminary results show limitations in health-related quality of life (HRQoL). The aim of this study is to show a possible change depending on the duration since infection and the accumulation of symptoms. Additionally, other possible influencing factors will be analyzed.

**Methods:**

The study population consisted of patients (18–65 years) presenting to the Post-COVID outpatient clinic of the University Hospital Jena, Germany, between March and October 2021. The HRQoL was assessed by the use of the RehabNeQ and the SF-36. Data analysis was descriptive with frequencies, means, and/or percentages. In addition, a univariate analysis of variance was performed to show the dependence of physical and psychological HRQoL on specific factors. This was finally tested for significance at an alpha level of 5%.

**Results:**

Data from 318 patients were analyzed, most of whom had 3–6 months of infection (56%) and 5–10 symptoms persisted (60.4%). Both mental (MCS) and physical sum score (PCS) of HRQoL were significantly lower than those of the German normal population (*p* < .001). The number of remaining symptoms (MCS *p* = .0034, PCS *p* = .000) as well as the perceived ability to work (MCS *p* = .007, PCS *p* = .000) influenced the HRQoL.

**Conclusion:**

The HRQoL of patients with Post-COVID-syndrome is still reduced months after infection and so is their occupational performance. In particular, the number of symptoms could have an influence on this deficit, which would need to be further investigated. Further research is needed to detect other factors influencing HRQoL and to implement appropriate therapeutic interventions.

## Background

The pandemic, which has been going on for almost two years now and is caused by the new SARS-COV-2 virus, has more and more influence, also on people who are not ill and not infected. High loads in health service, increased care expenditure of children at home and the partial long isolation of friends and relatives is accompanied with impairments for all parts of the population [1–3].

For individuals who have experienced COVID-19-disease, there may be additional health-related problems. A number of studies have now shown that infection with the SARS-CoV-2 virus can lead to severe acute respiratory syndrome (SARS) and long-lasting symptoms [4–9]. The cause for the development of a so-called Post-COVID syndrome is still unclear, but recent evidence suggests a possible association with sustained activation of pulmonary endothelial cells as well as the entire immune system, and aberrant immune responses or autoimmunity are also postulated [10–12]. Moreover, an internationally recognized naming of the persistent symptoms does not yet exist. However, a system is increasingly emerging that designates occurring symptoms 4 up to 12 weeks after the acute illness with “Long-COVID” and symptoms that persist over a period of more than 12 weeks with “Post-COVID-syndrome” [13, 14]. Because 98.1% of the cohort presented here were interviewed more than 12 weeks after infection, the term “Post-COVID-syndrome” is used.

In addition to the development of therapeutic concepts and the assessment of needs of the affected persons, the restoration of social and occupational participation of the large number of patients must also be taken into account [15–17]. Many sufferers are unable to work for months after infection, with both socioeconomic and individual consequences [18]. This can have influences on health-related quality of life (HRQoL).

Longer-lasting symptoms after acute infections with coronaviruses are also known in Middle East respiratory syndrome and other forms of SARS [19–21]. These include, similar to Post-COVID-syndrome, fatigue, muscle pain, and mental dysfunction [20–22]. Research have shown that HRQoL decreases further as the disease progresses [19]. Hui et al. studied recovered patients after hospitalized SARS for 3, 6, and 12 months after symptom onset. They showed that some scores of the HRQoL questionnaire (SF-36) improved over time (role physical, social function, role emotional) while others showed no change over time (physical function, bodily pain, vitality and mental health) and remained below normal values until the end. The general health score even showed a significant deterioration over time. A significant difference after 12 months between patients who were treated in an intensive care unit and those who did not require this therapy could not be found [23]. Influenza virus infections (H1N1) can also lead to acute respiratory distress syndromes (ARDS) [24, 25]. Studies on HRQoL showed that quality of life continued to increase 6 months after discharge from the hospital and that even patients who required intensive care therapy returned to approximately the values of the normal population after one year [24, 25]. Irrespective of the pathogen, patients with ARDS treated with intensive care continued to show limitations in functioning and HRQoL even five years after critical illness [26]. Consequently, the development of a post-intensive care syndrome may be considered to be the cause of the limitations [27]. To our knowledge, there are no data on HRQoL for mild courses of influenza, as no long-term symptoms occur.

Therefore, the question arises to what extent persistent symptoms after SARS-CoV-2 virus infection also lead to a reduction in HRQoL, both in hospitalized and non-hospitalized patients. Malik et al. conducted a systematic review with meta-analysis on this question. A total of twelve studies were included with a total of 4828 patients who reported continued symptoms after a period of up to 180 days after infection. 558 of the patients studied received intensive care. The prevalence of impaired quality of life measured by the visual analogue scale (EQ-VAS) was 59% across the entire sample. Furthermore, it was shown that patients who received intensive care and had fatigue as an existing symptom had a significantly worse outcome than those to whom these two things did not apply [28]. In addition, Meys et al. showed that HRQoL values are still below normal population levels three months after infection, even in non-hospitalized patients. [29]

This raises the question of whether patients who had COVID-19 and still have symptoms of Post-COVID syndrome are affected differently in their health-related quality of life depending on how long ago the infection occurred. In addition, the aim of the study is to analyse predictors that may affect HRQol. These include ability to work, number of existing symptoms, and received therapies.

## Methods

The cross sectional study included all patients presenting to the Post-COVID outpatient clinic at Jena University Hospital between first of March and 29th October 2021. Each patient completed the SF-36 and the COVID-19 Rehabilitation Needs Questionnaire (RehabNeQ) as part of the structured baseline assessments described elsewhere [17, 30–32]. The SF-36 is a valid and reliable assessment tool for HRQoL with reliability coefficients above 0.70 for each subscale and even above 0.80 for the summary scales [33, 34]. The RehabNeQ, a structured self-report assessment of COVID-related health information, is also reliable by means of dichotomous answer options of the present cohort with a reliability coefficient of 0.6 or higher (Kuder-Richardson reliability coefficient) [35]. As part of the assessment described, patients consented to the use of their data for research purposes. The only condition for an appointment was to have had PCR-confirmed SARS-CoV-2 infection.

Additional inclusion criteria were as follows:

- Age between 18 and 65 years
- Ability to work before COVID-19

The following data of the RehabNeQ were extracted:

- Time since infection
  The period since infection was calculated from the date of infection and the date of treatment. Subsequently, categorization into up to 3 months, 3- 6 months, 6-12 months or more than 12 months was performed.
- Symptoms still present
  From a list of 14 symptoms (sleep disturbance, intestinal dysfunction, restriction of movement, muscular problems, Problems of the respiratory tract, limitations of sense of smell, limitation of sense of taste, vascular occlusion, fatigue, hair loss, memory disorders, tinnitus, dizziness, pain) patients selected those that were still present at the time of the assessment.
- Therapies received
  This category of questions included individual questions with the answer options yes/no. In terms of content, the questions focused on the therapies received and the subjective feeling of having had difficulties in receiving them.
- Ability to work (real and perceived)
  The current work ability was assessed by means of a selection question. For the perceived work ability, the first question of the Work Ability Index was used, which reflects the perceived work ability on a 10-step scale (0 unable to work - 10 best able to work) [36].
- Inpatient treatment
  The question about inpatient treatment during the acute phase of COVID-19 disease was recorded by yes/no question.
- Sociodemographic data (age, sex, marital status).

### Statistical analysis

After positive ethical vote of the University Hospital of Jena (2021–2424-Daten), descriptive analysis was performed by presenting frequencies, means and percentages. In addition, the results of the SF-36 were compared with the German Norm Data by use of the Students-T-Test. Furthermore, a general linear model, and here in concrete the univariate analysis of variance was used to illustrate the dependence of the health-related quality of life by means of the SF-36 (physical and psychological HRQoL) on different parameters. As fixed factors gender, the time of infection, the number of symptoms and the difficulties in getting the needed therapies were set. Because of the metric character, only the factor age was set as covariate. The analysis of variance was used because of the primary nominal and ordinal scaled level of measurement of the independent variables. This was finally tested for significance at an alpha level of 5%. As effect size, the partial Eta squared was calculated. For this purpose, the statistical program IBM SPSS Statistics (version 26) was used.

## Results

384 patients presented to the Post-COVID outpatient clinic at Jena University Hospital within the study period, 336 of them met the inclusion criteria, 46 patients were excluded, as they were older than 65 years, 2 patients were already unable to work before COVID-19.

**Fig. 1:**
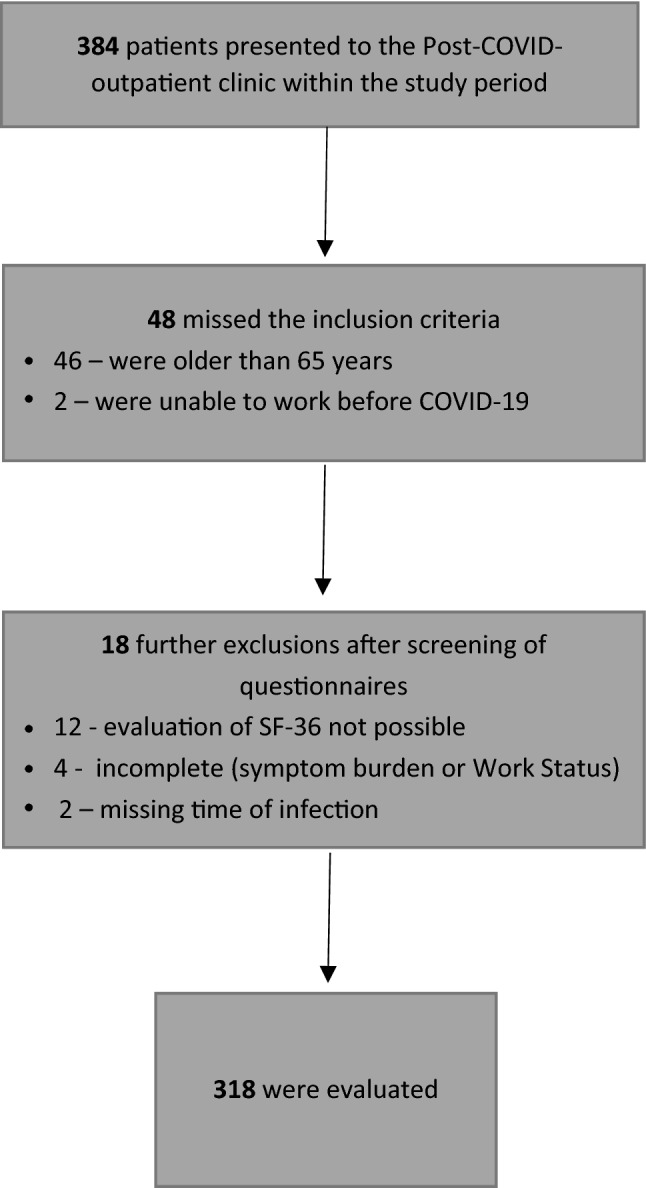
Flow chart for the course of study

After reviewing the questionnaires, further exclusions of 18 individuals were made because the SF-36 was not completed (*n* = 12), too much information was missing in the area of symptom burden or statements about work ability (*n* = 4), or the time since infection was not reported (*n* = 2). Thus, in conclusion, 318 patients were included whose questionnaires were fully completed (detailed information is shown in Fig. 1).

68.9% (*n* = 219) of the included patients reported being female and were on average 46.9 (± 10,9) years old. 56.9% (*n* = 181) of the respondents were married. 18.6% (*n* = 59) were living in a partnership. The information on other forms of family status was distributed among single (13.2%, *n* = 42), separated/divorced (8.5%, *n* = 27) and widowed (1.9%, *n* = 6). 2 respondents did not answer.

The majority of respondents reported having been infected with the SARS-CoV-2 virus between 6 and 12 months ago (56.0%, *n* = 178). In some cases, the time of infection was already more than 12 months ago (14.8%, *n* = 47). A total of 93 persons indicated periods of up to 6 months since infection, of which 87 (27.4%) indicated 3 or more months and 6 (1.9%) less than 3 months. Of the respondents who participated in the study, 75 (23.6%) stated that they had been hospitalized. Of these, 13 (17.3%) persons had received mechanical ventilation. The correlation with the time of infection is shown in Table 1

**Table 1:** Overview of work ability (perceived and real) and existing symptoms in relation to the time period since infection (answers from RehabNeQ)

Work ability	Time since infection			
	< 3 months	≥ 3 months	≥ 6 months	≥ 12 months
•. Patients hospitalized due to COVID-19 (*n* = 75)	1 (16.7%)	23 (26.4%)	38 (21.3%)	13 (27.7%)
•. Patients unable to work at the time of the survey (*n* = 126)	4 (3.2%)	44 (34.9%)	65 (51.6%)	13 (10.3%)
•. Average perceived ability to work in points (max. 10) (*n* = 190)	5.5 (± 2.1)	6.1 (± 1.9)	5.9 (± 1.8)	6.7 (± 1.8)
•. Average number of existing symptoms (max. 14) (*n* = 318)	7.8 (± 3,7)	5.7 (± 2,4)	5.9 (± 2,6)	5.7 (± 2,8)

### Ability to work

190 (59.7%) of the included persons stated that they were able to work at the time of the survey. 126 (39.6%) were current unable to work due to COVID 19 disease, 89 (70.6%) of them continuously since the onset of the infection. Two persons have meanwhile entered the old-age pension. The percentage distribution of those unable to work in relation to the duration since the infection is shown in Table 1.

When asked about perceived work ability, where 0 points means “unable to work” and 10 points means “best achieved work ability,” respondents who were able to work answered with 6.1 (± 1,9) points on average. Female respondents gave themselves a below average score (5.9 ± 1,9). The relationship between perceived ability to work and time of infection is shown in Table 1.

### Symptoms

In the symptoms section, 14 symptoms were provided for selection. The most common symptoms were still fatigue (83.3%, *n* = 265), sleep disturbances (69.8%, *n* = 222) and mental disorders (67.0%, *n* = 213). The majority (60.4%, *n* = 192) of patients still had 5 to 10 symptoms at the time of the survey.

The correlation between existing symptoms and time of infection is shown in Table 1.

### Therapy

176 (55.3%) of the respondents answered „yes” concerning the question whether they had difficulties to receive appropriate therapies. 92 people used the free text field to give further reasons. Of these, 73 (79.3%) referred to problems with the treating doctor. These included the lack of knowledge, the difficulty of getting appropriate appointments, the feeling that they were not taken seriously or that they should have more patience.

119 (67.4% of those who reported difficulties in receiving therapies) persons answered the multiple-answer question about therapy wishes with the wish for a contact person or offers of talks. 86 (48.9%) would have liked to have outpatient therapies,49 (27.3%) inpatient or outpatient rehabilitation treatments.

### SF-36

The summed results of the SF36 of the total group, also in comparison to the German healthy normal population, are shown in Table 2 [31]. In all items, the cohort shows significantly lower values than the normal population (*p* < 0.001). The averaged component summary scores of the respondents are reduced by 12.1 points (physical component summary, PCS) and 10 points (mental component summary, MCS) compared to the German standard values. Overall, 66% of respondents showed limitations in PCS and 50.6% in MCS.

**Table 2:** Results of SF-36 itemized by the single dimensions, entire study group in comparison to German norm data (*n* = 318) [31]

	PF	RP	BP	GH	VT	SF	RE	MH	PCS	MCS
*n* = 318 mean (± SD)	57.2 (± 24.4)*	26.9 (± 33.1)*	51.6 (± 29.1)*	45.8 (± 18.8)*	33.7 (± 19.8)*	54.0 (± 26.9)*	49.8 (± 45.0)*	59.2 ( ± 19.5)*	36.3 (± 10.1)*	40.9 (± 11.6)*
German standard values [31]	85.4 2 (± 20.7)	82.4 (± 32.7)	67.4 (± 25.9)	66.4 (± 18.2)	60.0 (± 17.8)	86.4 (± 19.9)	89.1 (± 26.7)	72.5.1 (± 16.7)	48.4 (± 9.4)	50.9 (± 8.8)

*PF* physical functioning, *RP* role physical, *BP* bodily pain, *GH* general health, *VT* vitality, *SF* social functioning, *RE* role emotional, *MH* mental health, *PCS* physical component summary, *MCS* mental component summary

*Sign (*p* < .001))

Table 3 shows the dependence of variances related to time since infection, number of symptoms, the difficulty receiving therapies, and perceived ability to work. Moreover, age and gender as possible confounders are included, too. In both PCS and MCS, the number of symptoms (up to 14) and perceived ability to work have a significant impact on HRQoL assessed by the SF-36. However, the effect concerning the PCS can be interpreted as high, whereas the effect on MCS is moderate. No influence has the duration since the infection, as well as the not receiving of desired therapy sessions. Age and gender show no significant influence here.

**Table 3:** Results of the univariate analysis of covariance concerning the fixed factors in relation to the SF-36 sum scores

		Covariate (age) and further fixed factors	Regression coefficient (95%-CI)	df	*F* value	*p* value	Partial eta^2^
SF-36	PCS	Age (in years)	− .028 (− .110 to .55)	1	.437	.509	.000
		Gender (female vs. male)	− .331 (− 2.251 to 1.589)	1	.115	.735	.002
		Time since infection		3	1.145	.331	.013
		< 3 months vs. ≥ 12 months	− 4.092 (− 10.705 to 2.520)				
		≥ 3 months vs. ≥ 12 months	.122 (− 2.801 to 3.045)				
		≥ 6 months vs. ≥ 12 months	1.051 (− 1.614 to 3.716)				
		Number of symptoms		12	3.159	.000*	.128
		1 vs. 13	12.16(2.026 to 22.186)				
		2 vs. 13	3.711 (− 5.774 to 13.196)				
		3 vs. 13	7.126 (− 1.894 to 16.146)				
		4 vs. 13	5.384 (− 3.660 to 14.428)				
		5 vs. 13	5.642 (− 3.330 to 14.613)				
		6 vs. 13	7.058 (− 1.838 to 15.955)				
		7 vs. 13	2.651 (− 6.270 to 11.572)				
		8 vs. 13	1.846 (− 7.113 to 10.805)				
		9 vs. 13	1.334 (− 7.980 to 10.648)				
		10 vs. 13	2.638 (− 6.879 to 12.154)				
		11 vs. 13	− 3.700 (− 13.540 to 6.141)				
		12 vs. 13	− 1.109 (− 11.917 to 9.699)				
		Perceived ability to work		10	10.849	.000*	.295
		0 vs. 10	− 20.920 (− 31.724 to − 10.116)				
		1 vs. 10	− 23.535 (− 38.652 to − 8.418)				
		2 vs. 10	− 25.694 (− 40.697 to − 10.691)				
		3 vs. 10	− 22.617 (− 34.522 to − 10.713)				
		4 vs. 10	− 18.659 (− 29.857 to − 7.461)				
		5 vs. 10	− 15.209 (− 26.322 to − 4.095)				
		6 vs. 10	− 15.583 (− 26.545 to − 4.620)				
		7 vs. 10	− 10.458 (− 21.368 to .451)				
		8 vs. 10	− 10.711 (− 21.520 to .097)				
		9 vs. 10	− 4.300 (− 15.487 to 6.887)				
		Difficulties to receive therapies (yes vs. no)	− .904 (− 2.808 to 1.001)	1	0.873	.351	.003
	MCS	Age (in years)	− 0.35 (− .154 to .083)	1	.346	.557	.001
		Gender (female vs. male)	− .099 (− 2.856 to 2.658)	1	.005	.943	.000
		Time since infection		3	.938	.423	.011
		< 3 months vs. ≥ 12 months	3.541 (− 5.953 to 13.036)				
		≥ 3 months vs. ≥ 12 months	− 2.597 (− 6.794 to 1.601)				
		≥ 6 months vs. ≥ 12 months	− 1.746 (− 5.572 to 2.081)				
		Number of symptoms		12	1.859	.040*	.079
		1 vs. 13	14.126 (− .348 to 28.599)				
		2 vs. 13	18.362 (4.743 to 31.981)				
		3 vs. 13	17.679 (4.727 to 30.630)				
		4 vs. 13	15.451 (2.465 to 28.437)				
		5 vs. 13	15.160 (2.278 to 28.041)				
		6 vs. 13	14.341 (1.567 to 27.116)				
		7 vs. 13	13.989 (1.180 to 26.798)				
		8 vs. 13	13.405 (.541 to 26.269)				
		9 vs. 13	9.430 (− 3.943 to 22.804)				
		10 vs. 13	17.800 (4.136 to 31.464)				
		11 vs. 13	17.344 (3.214 to 31.474)				
		12 vs. 13	2.496 (− 13.023 to 18.015)				
		Perceived ability to work		10	2.446	.008*	.086
		0 vs. 10	− 18.124 (− 33.636 to − 2.611)				
		1 vs. 10	− 33.734 (− 55.440 to − 12.028)				
		2 vs. 10	− 16.587 (− 38.129 to 4.956)				
		3 vs. 10	− 23.613 (− 40.707 to − 6.519)				
		4 vs. 10	− 18.139 (− 34.21 to − 2.060)				
		5 vs. 10	− 16.173 (− 32.131 to − .216)				
		6 vs. 10	− 18.238 (− 33.979 to − 2.497)				
		7 vs. 10	− 14.366 (− 30.031 to 1.298)				
		8 vs. 10	− 11.996 (− 27.516 to 3.524)				
		9 vs. 10	− 8.625 (− 24.689 to 7.438)				
		Difficulties to receive therapies (yes vs. no)	− 1.553 (− 4.287 to 1.182)	1	1.250	.265	.005

*PCS* Physical Component Summary, *MCS* Mental Component Summary, *df* degrees of freedom

*Significant

**Fig. 2:**
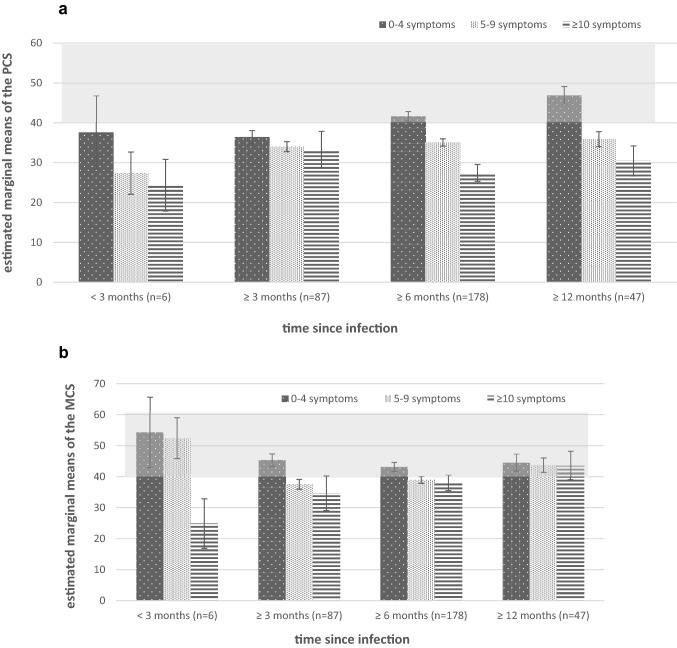
**a** Effect of the time of infection and the number of symptoms on Physical Component Scale (PCS) (range of symptoms: 1 = 0–4 symptoms, 2 = 5–9 symptoms, 3 = 10–14 symptoms, grey coloured area = normal range). **b** Effect of the time of infection and the number of symptoms on Mental Component Scale (MCS) (range of symptoms: 1 = 0–4 symptoms, 2 = 5–9 symptoms, 3 = 10–14 symptoms, grey coloured area = normal range)

The influence of the number of symptoms on physical (PCS, Fig. 2a) and mental (MCS, Fig. 2b) sum score differs. The PCS and MCS value of 50 ± 10 is considered as standard value (grey highlighted in Fig. 2a and b). In this study the PCS mean descends with the increasing number of symptoms, largely independent of the time since the infection started. However, the MCS mean achieves the standard level again after a longer period of infection, but even with a high number of symptoms.

Being hospitalized has apparently no influence on physical or mental health for there seems to be no difference between inpatients or outpatients regarding the means based on standard value and existing deficits (Table 4). The distribution appears similar because while 77% of the inpatients complained a reduced PCS, 62% of the outpatient stated a deficit. Even more obviously it is regarding mental health because 52% of the inpatients experience a minor health level while exactly the half of the outpatient study population feels mentally restricted (according to a significance level of 5%).

**Table 4:** Distribution of quality of life in physical (PCS) and mental (MCS) health with regard on hospital stay

Number of patients (n)	PCS		MCS	
	German norm	deficit	German norm	Deficit
Inpatient/hospitalized	17	58	36	39
Outpatient/not-hospitalized	91	151	121	121

*PCS* Physical Component Summary, *MCS* Mental Component Summary)

Consideration of the current work ability at the time of the survey showed that those who were unable to work (*n* = 126) showed deficits in the majority in both sum scores (PCS 88.1%, MCS 58.7%). Respondents who were able to work (*n* = 190) showed in the majority deficits in the PCS (51.6%, *n* = 98), but not in the MCS (45.7%, *n* = 87).

## Discussion

The study provides indications that especially the number of remaining symptoms with regard to the post-COVID syndrome has an impact on HRQoL. However, unlike Malik et al., there was no difference between hospitalized and non-hospitalized patients in the acute infection phase. Nevertheless, it should be noted that in the sample studied, only a very small proportion was severely ill and therefore required inpatient hospitalization. In addition, the analysis showed that there is a correlation between the perceived ability to work and the quality of life, which is comprehensible. The overall cohort showed a significant reduction in HRQoL compared to the normal German population [31]. This was also independent of the duration of infection. A reduction in HRQoL after COVID-19 disease has also been demonstrated by other authors, although the number of studies is still very small, especially in the longer follow-up [28, 37]. Poudel et al. could show in the three analysed studies, which included patients with a time since infection of more than four weeks, that the values of the PCS were slightly higher than those of the MCS [37]. This is contrary to our results. Reasons may lie in the smaller sample or in the different long follow-up (Poudel et al., max 12 weeks follow up). Also, the composition of the sample could provide an explanation, as it was especially the older (> 65 years) patients who showed particularly strong deficits in HRQoL according to Poudel et al. For this study, only patients of working age (up to 65 years) were included. Finally, the time of the survey could also have an influence. The studies included by Poudel et al. were collected in 2020 [37]. The cohort of this sample became infected for the most part at the end of 2020 until May/June 2021. In March, the delta variant of the SARS-CoV-2 virus was detected for the first time in a polymerase chain reaction test in Germany. Compared to the wild type, slightly altered symptoms were observed in non-research publication during the acute course [38, 39]. Whether this also applies to the Post-COVID syndrome remains to be seen and should be investigated in more detail.

The symptoms most frequently reported by respondents were fatigue, sleep disturbances and memory impairment, similar to other studies [6, 17, 40, 41]. These may influence HRQoL, although other pandemic-related aspects, such as loss of social contacts, job insecurities, and limitations in daily life, should not be ignored in this consideration [42].

The analysis of the data presented here showed that the values of the MCS of those affected, in whom the infection has already occurred more than 12 months ago, are again at the physiological standard level. It is interesting to note that this is independent of the number of symptoms still present.

Thus, there seems to be adaptation through coping skills, self-efficacy as a way to maintain a psychological “steady state”. Further research on how a patient recalibrates their self-assessment of health-related quality of life or their cognitive appraisal processes is needed [43]. In contrast, the number of existing symptoms seems to have more of an influence on the physical aspects of the HRQoL. Only a few lingering symptoms show a return to normal levels in affected individuals with at least 6 months interval from infection. The greater the number of symptoms, the worse the scores in the PCS. A limitation in function and participation, and thus a rehabilitation need, can therefore be postulated [7, 8].

Overall, however, it appears that there may be improvement in psychological and physical function over the course of one year, similar to Huang et al. [42]. Comparable analyses in survivors of SARS still showed limitations in almost all SF-36 scales at both 12 and 24 months [23, 44]. How HRQoL develops in patients with Post-COVID syndrome remains to be seen and should be studied in detail scientifically. This is necessary according to our results with special focus on the still existing symptoms. How the number of symptoms and their severity develops in the course of the disease is still unclear, although an improvement of some of them can be assumed, which is also shown by our data [42]. Davis et al. showed similar results [45]. He divided the existing symptoms into three clusters: Cluster 1: Symptoms that are very frequent at the beginning of the infection and occur less frequently over time (e.g. dry cough, fever). Cluster 2: Symptoms that are relatively stable in their probability of occurrence over a period of up to 7 months (e.g. muscle aches, shortness of breath, dizziness) and cluster 3 with symptoms where the probability of occurrence only increases in the course of the disease and the further course is variable (e.g. brain fog, tinnitus, palpitations) [45]. A variability of the temporal course must therefore be assumed. A meta-analysis showed that from the 60th day after infection there was an increase in symptom burden [40]. Here it is necessary to observe the course of the severity of individual symptoms over a longer period of time and to investigate which influences the patients are exposed to.

The study was also able to show that HRQoL correlates with real and perceived incapacity to work. Almost 40% of the respondents stated that they were unable to work, as was already found in other studies at a similar level [18]. 70% of them were even permanently unable since the infection and thus partly more than 1 year. Although our results, similar to those of Huang et al., show that between the 6th and 12th month after infection there is often a return to work, however, the perceived ability to work increases only slightly in comparison [42]. A restriction in work performance therefore remains. This is also confirmed once again by the results of this analysis. More than half of those able to work have deficits in the PCS of the HRQoL. Therefore, it can be assumed that the productivity of these present employees is reduced by the still existing health problems. The cost of this is similar to that of actual absenteeism [46]. In contrast to the deficits in the physical sum scale of those able to work, the values of the mental sum scale were mostly within the normal range. This could indicate that mental health is more important than physical health for the ability to work. A survey of employed persons in Germany showed that mental stressors in particular were reported by around 30% of respondents. A high level of concentration and attention is required to cope with these tasks [47]. Restrictions, such as concentration disorders, stand in the way of this and thus also of returning to work. According to our analysis, the majority of patients who are unable to work still show deficits in both scales. Economic aspects, such as continued payment of wages in the event of illness, must also be taken into account in this analysis. The financial losses caused by long absences are not bearable for some in the long run, so that mental recovery, as a first step, could have an important influence here [48–50]. However, there is no data yet on this particular angle of view, how it affects patients with Post-COVID syndrome.

More than half of the respondents reported problems obtaining outpatient therapies. Although the analysis could not show that this has an influence on HRQoL, Therapeutic interventions, also in the context of multidisciplinary phase-specific (tele-) rehabilitative measures, are necessary to alleviate symptom severity and to promote activity and participation again [51–53]. The clinics and therapists must be prepared and specially trained for this [54, 55].

### Limitations

The results of this study have to be considered with some limitations. The queried symptoms were not clinically objectified, so that here a disproportionate indication can be displayed. In addition, selection bias cannot be ruled out, since this is a cohort that presented to a specialized consultation. There is no comparison to patients who presented only to primary care physicians. The excess of female respondents has already been reported in other studies on the development of Post-COVID syndrome, but it must also be added that men in general seek less medical help than women, which could exacerbate this imbalance [40, 56, 57].

In addition, the individual persons were not observed over the entire period of time and thus individual changes were not considered. Furthermore, information on physical and mental disorders prior to the COVID-19 disease, which may have already influenced the quality of life before, was not requested. Further research including these more individual aspects is required to identify factors that may contribute to a positive development of HRQoL. When interpreting the data, it is important to note that the comparison of HRQoL presented here was made with the normal German population before the pandemic [31]. Many studies have recently shown that pandemic-specific requirements and restrictions, such as lockdown or social distancing, can lead to changes in health-related quality of life, even in uninfected individuals [58–60]. A Danish study showed significant changes in both MCS and PCS, with women more affected than men [61]. For Germany, an app-based study of nearly 1400 respondents was able to show that there was already a reduction in both mental and physical health-related quality of life, as measured by the World Health Organization Quality of Life Questionnaire, between the mid- and late-2020s.[62]. Current data collected with the SF-36 for comparison with the cohort shown are not yet available for Germany. International data are not valid comparators and a measure of the change in the overall mental health (MCS) and physical health from Germany would be a more appropriate control group rather the Pre COVID German population norms. As such our results may overestimate the effect COVID-19 disease has compared to a control group who are living in the same environment with COVID lockdowns and similar environmental stressors.Future studies should take this aspect into account and investigate the differences in quality of life between COVID-19-infected and non-infected patients in large samples.

## In conclusion

The HRQoL of patients with Post-COVID syndrome is reduced for months after infection, and so is their occupational performance. The presented normalization of the subdomains of HRQoL and the reduction of the remaining symptoms gives hope for the recovery of the patients and the return to the baseline level of activities and participation that existed before the COVID-19 disease. Further research in this area is needed to detect further positive or negative factors influencing HRQoL. Subgroups should also be considered. The results should be used to optimize therapeutic rehabilitative measures. A consideration of the symptoms depending on the virus variant leading to the infection may help to explain differences in studies and to establish therapy approaches even more individual.

## Data Availability

The datasets generated during and/or analysed during the current study are available from the corresponding author on reasonable request.
